# G protein-coupled receptors function as cell membrane receptors for the steroid hormone 20-hydroxyecdysone

**DOI:** 10.1186/s12964-020-00620-y

**Published:** 2020-09-09

**Authors:** Xiao-Fan Zhao

**Affiliations:** Shandong Provincial Key Laboratory of Animal Cells and Developmental Biology, School of Life Sciences, Shandong University, Qingdao, 266237 China

**Keywords:** GPCR, Steroid hormone, 20-hydroxyecdysone, Cell membrane receptor, Signal pathway

## Abstract

**Abstract:**

G protein-coupled receptors (GPCRs) are cell membrane receptors for various ligands. Recent studies have suggested that GPCRs transmit animal steroid hormone signals. Certain GPCRs have been shown to bind steroid hormones, for example, G protein-coupled estrogen receptor 1 (GPER1) binds estrogen in humans, and *Drosophila* dopamine/ecdysteroid receptor (DopEcR) binds the molting hormone 20-hydroxyecdysone (20E) in insects. This review summarizes the research progress on GPCRs as animal steroid hormone cell membrane receptors, including the nuclear and cell membrane receptors of steroid hormones in mammals and insects, the 20E signaling cascade via GPCRs, termination of 20E signaling, and the relationship between genomic action and the nongenomic action of 20E. Studies indicate that 20E induces a signal via GPCRs to regulate rapid cellular responses, including rapid Ca^2+^ release from the endoplasmic reticulum and influx from the extracellular medium, as well as rapid protein phosphorylation and subcellular translocation. 20E via the GPCR/Ca^2+^/PKC/signaling axis and the GPCR/cAMP/PKA-signaling axis regulates gene transcription by adjusting transcription complex formation and DNA binding activity. GPCRs can bind 20E in the cell membrane and after being isolated, suggesting GPCRs as cell membrane receptors of 20E. This review deepens our understanding of GPCRs as steroid hormone cell membrane receptors and the GPCR-mediated signaling pathway of 20E (20E-GPCR pathway), which will promote further study of steroid hormone signaling via GPCRs, and presents GPCRs as targets to explore new pharmaceutical materials to treat steroid hormone-related diseases or control pest insects.

Video abstract

**Graphical abstract:**

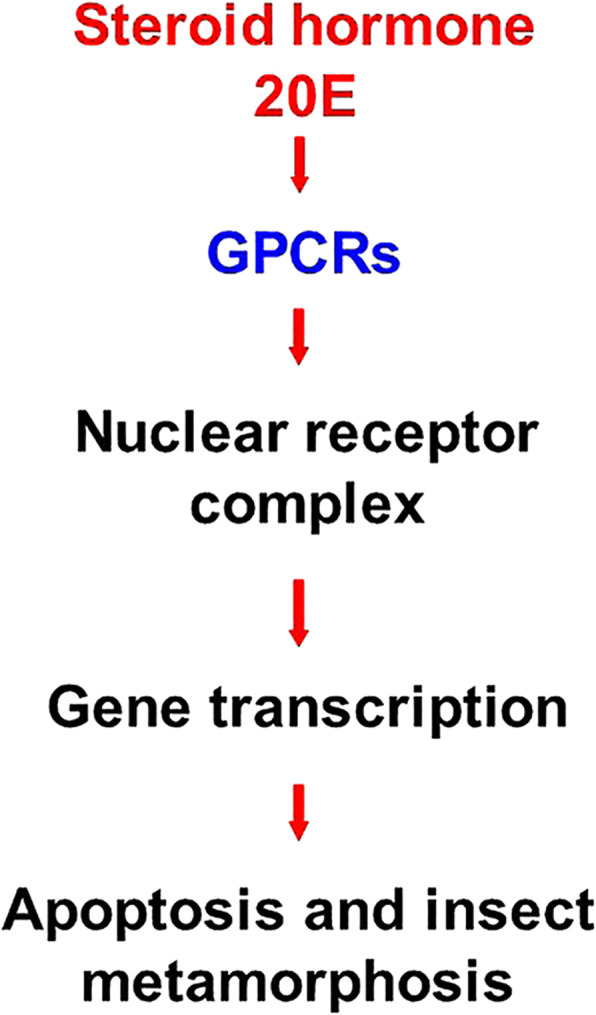

## Background

G protein-coupled receptors (GPCRs) are seven-transmembrane proteins that are located in the cell membrane, with their N- and C-termini located on the outer and inner surfaces, respectively. GPCRs mediate various cellular responses from the extracellular environment. A GPCR is activated upon binding of its ligand, which causes a conformational change in the GPCR’s structure. The activated GPCR then interacts with G protein to induce further signaling cascades [1]. Over 800 GPCRs have been identified in the human genome [2], 1000 in the *Caenorhabditis elegans* genome [3], and 200 in the *Drosophila melanogaster* genome [4]. GPCRs have been identified as the cell membrane receptors of various ligands, including biological amines, amino acids, ions, lipids, peptides/proteins, light, odorant, pheromones, nucleotides, and opiates [5]. However, the functions and pathways of GPCRs as receptors of animal, including insect steroid hormones have not been fully determined. This present review integrates evidence obtained from insects and mammals to demonstrate that GPCRs function as cell membrane receptors of animal steroid hormones.

## Steroid hormones and their nuclear and cell membrane receptors in mammals

Steroids are small lipophilic organic molecules with a four-ring structure, which are found in animals, plants, and fungi. The steroid cholesterol is an essential component of animal cell membranes, where it maintains membrane structure and fluidity. Most steroids function as signaling molecules, such as hormones [6]. Animal steroid hormones include estrogens, androgens, glucocorticoids, mineralocorticoids, and progestogens. Steroid hormones play vital roles in various processes in humans and animals. Thus, understanding the signaling pathways of steroid hormones is very important.

Animal steroid hormones are known exert their actions via binding to their intracellular nuclear receptors [7], for example, estrogen binds to its nuclear estrogen receptors ERα and ERβ [8], androgens bind to androgen receptors (AR) [9], and glucocorticoids bind to glucocorticoid receptors [10]. However, the plant steroid hormones, brassinosteroids, initiate signaling by combining with plasma membrane receptors [11]. The structural similarities between plant steroid hormones and animal steroid hormones [12] have led researchers to investigate the cell membrane receptors for animal steroid hormones.

Extensive evidence indicates that animal steroids activate receptors on the cell membrane. Steroid hormone action in musculoskeletal cells involves membrane receptors and the rapid cellular responses to regulate gene expression via signaling cascades [13]. However, these rapid cellular responses do not rely on gene expression and are therefore designated as nongenomic responses to distinguish them from the genomic responses that are based on gene transcription. For example, estrogen activates phosphoinositide 3 kinase (PI3K) to recruit protein kinase B (AKT/PKB) to the cell membrane in mammals via a mechanism independent of the genomic actions of hormones [14]. Pregnenolone, the precursor of androgens, estrogens, progesterone, mineralocorticoids, and glucocorticoids [15], regulates gene expression via a nuclear receptor-mediated genomic pathway and via a transient receptor potential (TRP) cation channels-mediated nongenomic pathway [16]. Several GPCRs are reported as progesterone receptors (mPRα, mPRβ, mPRγ, mPRδ, and mPRε) [17]. The cell membrane receptors of progestin have been identified in vertebrates [17, 18]. Androgen transmits signals via cell membrane receptors [19], which are distinct from the androgen nuclear receptors [20]. A zinc influx transporter (ZIP9), which is not a GPCR, has been identified as a membrane androgen receptor [21]. Testosterone mediates nongenomic effects via a calcium and amino acid sensing GPCR (GPRC6A) [22]. The estrogen receptor GPCR (GPR30) [23], which transmits estrogen signals from the membrane [24], has been renamed as G protein-coupled estrogen receptor 1 (GPER1) [25]. Estrogen transmits signals via GPERs to transactivate epidermal growth factor receptors for cell proliferation in female reproductive cancers [26]. GPER1 is reportedly located in the endoplasmic reticulum, but might translocate to the cell membrane [27]. Recent studies have revealed that GPER is constitutively internalized in an arrestin-independent manner and does not recycle to the cell membrane for further signaling [28]. GPER1-mediated nongenomic activity is independent of the estrogen nuclear receptor [26]. In addition to its function in estrogen signaling, GPER1 also functions in other biological systems, such as the nervous system to mediate neuroprotection; therefore, GPER1 is considered to be a pharmacological target [29, 30]. The identification of GPER1 opens a new field of research [31]. Accumulating evidences supports membrane-initiated estrogen signaling [32]. However, these non-classical steroid actions are not widely accepted and littles progress has been made since the discovery of rapid steroid hormone actions in the 1980s [33]. Identification of the steroid ligands of GPCRs represents a major challenge for studies of the steroid hormone nongenomic pathways [34].

## 20-hydroxyecdysone and its nuclear and cell membrane receptors in insects

20-hydroxyecdysone (20E), which is also known as the insect molting hormone, initiates insect larval molting from one instar to the next (molting), or the metamorphic molting from larva to adult (metamorphosis) [35, 36]. Similar to other animal steroid hormones, 20E is thought to diffuse freely into cells because it is a fat-soluble molecule. 20E binds to its nuclear receptor, ecdysone receptor (EcR), to exert its effect on gene transcription in the classical genomic pathway. EcR must interact with the ultraspiracle protein (USP), retinoid X receptor (RXR) in Hemimetabola, the ortholog of the retinoid X receptor in vertebrates, to form a heterodimeric transcription complex, EcR/USP [37]. This complex binds to ecdysone response elements (EcRE) to regulate 20E-responsive gene transcription [36], such as hormone receptor 3 (HR3), an early-late gene in the 20E pathway [38].

The earlier evidence that 20E triggers rapid nongenomic actions before gene transcription was obtained in studies of the anterior silk gland of *Bombyx mori*. The plasma membrane can bind [^3^H] ponasterone A ([^3^H] Pon A), suggesting the existence of an unknown membrane receptor [39]. Other evidence is provided by the observation that 20E induces rapid increase of Ca^2+^ levels in the cells of the anterior silk gland of *B. mori* via an unknown GPCR pathway [40]. 20E also triggers rapid increased Ca^2+^ in mouse skeletal muscle cells via GPCRs [41]. However, the cell membrane receptor-mediated nongenomic pathway of 20E is not fully understood.

A GPCR, *Drosophila melanogaster* dopamine/ecdysteroid receptor (DmDopEcR), is considered a 20E cell membrane receptor in *Drosophila*. The supporting evidence includes the observation that the membrane of Sf9 cells that overexpress DmDopEcR could bind [^3^H] Pon A. In addition, 20E triggers intracellular rapid increases in Ca^2+^ and cyclic adenosine monophosphate (cAMP), and increases ERK phosphorylation in the DmDopEcR overexpressing Sf9 cells [42]. DmDopEcR functions as a 20E receptor to modulate the basal and acute physiology of brain structures and behavior [43]. *Agrotis ipsilon* DopEcR (AipsDopEcR) is predominantly expressed in the nervous system, including the mushroom bodies. AipsDopEcR is involved in the expression of sexual behavior in the male moth [44]. 20E and dopamine (DA), via AipsDopEcR, control sex pheromone perception in the central nervous system [45]. DopEcR plays a significant role in the rapid actions of steroids in a variety of biological processes, such as behavioral modulation in the nervous system [43]. DopEcR plays multiple functions in response to various stressors in *Drosophila* [46]. The evidence suggests that 20E transmits signals via cell membrane receptors and that a nongenomic pathway exists.

## The 20E signaling cascade via GPCRs

The 20E-responsive GPCR (initially designated ErGPCR, and later, ErGPCR-1) [47] and ErGPCR-2 [48] are further revealed in *Helicoverpa armigera*. ErGPCR-1 expression levels are increased at the molting and metamorphic stages under 20E regulation. ErGPCR-1 is essential for 20E pathway gene expression and larval-pupal transition. Overexpression of ErGPCR-1 in HaEpi cells (*H. armigera* epidermal cell line) increases 20E pathway-related gene expression. 20E induces a rapid increase in cytosolic Ca^2+^ levels and promotes calponin nuclear translocation and phosphorylation via ErGPCR-1 [47]. ErGPCR-2 has a similar function to ErGPCR-1, such as regulation of rapid increases in intracellular Ca^2+^ levels, and phosphorylation of USP [48]. The main difference is that ErGPCR-2 can be internalized from the cell membrane to the cytosol under 20E induction. After internalization, ErGPCR-2 is degraded by proteases to terminate 20E signaling. [^3^H] Pon A entry into cells relies on ErGPCR-2 localization in the cell membrane [48]. A recent study shows that ErGPCR-2 and DopEcR in *H. armigera* could bind 20E in the cell membrane or as isolated proteins, using a 20E enzyme immunoassay (20E-EIA). That study also demonstrates one of the mechanisms by which 20E represses larval feeding and promotes metamorphosis: 20E competes with dopamine to bind to DopEcR to block the dopamine-mediated motor function and reward-motivated behavior, and initiates the 20E pathway [49].

Both ErGPCR-1 and ErGPCR-2 belong to the Methuselah-2 GPCRs of the class B secretin family and are located in the cell membrane. However, ErGPCR-1 contains 489 amino acids with a 19-amino acid signal peptide, whereas ErGPCR-2 contains 757 amino acids without a signal peptide [48]. In contrast, DmDopEcR shows homology with vertebrate ARs in GPCR class A [42]. Phylogenetic analysis using amino acid sequences show that ErGPCR-1 and ErGPCR-2 differ from GPR30, beta-2 AR, or *Drosophila* DmDopEcR [48]. Studies on ErGPCR-1, ErGPCR-2, and DopEcR suggest the possibility that several GPCRs are involved in 20E signaling via the participation of several downstream cascades or the differential expression and distribution of GPCRs in tissues [49].

G proteins directly transmit GPCR signals [1]. In *H. armigera*, the phosphorylation of G protein alpha q subunit (Gαq) is induced by 20E [50]. Gαq is located in the cytoplasm in HaEpi cells and is induced to migrate toward the cell membrane by 20E. 20E induces Gαq protein kinase C (PKC)-phosphorylation and membrane trafficking. Gαq participates in the 20E-induced increase in intracellular Ca^2+^ levels and is necessary for larval development, metamorphosis, and 20E pathway gene expression, and plays roles downstream of ErGPCR-1 in 20E signaling [50]. Gαq directly activates phospholipase C β [51]. The mRNA levels of phospholipase C gamma 1 (PLCG1) are increased at the molting and metamorphic stages in *H. armigera* [52]. In that study, RNAi-mediated silencing of *PLCG1* blocks 20E-induced pupation, larval death, and pupation. 20E pathway-related gene expression is also repressed by *PLCG1* silencing. Studies in *H. armigera* demonstrate that the function of PLCG1 in the 20E signaling pathway is mediated via ErGPCR-1, Gαq, and Src-family kinases, and that 20E mediates tyrosine phosphorylation at the SH2 domain of PLCG1. Activated-PLCG1 migrates toward the cell membrane to initiate intracellular Ca^2+^ signaling and calcium channel-controlled Ca^2+^ influx, which triggers PKC-mediated USP phosphorylation to modulate USP binding to EcRE for subsequent gene transcription. These findings provide evidence that 20E regulates the genomic pathway for gene transcription through an ErGPCR-1/ Gαq/PLCG1/Ca^2+/^PKC-dependent nongenomic pathway [52].

Ca^2+^ ion is an important secondary signal messenger in cells. The concentration of Ca^2+^ is well controlled at low levels inside cells, but can be increased by influx from outside the cells by various signals [53]. After signaling, the intracellular Ca^2+^ is decreased by excluding Ca^2+^ out of cells and storing Ca^2+^ in the endoplasmic reticulum (ER) [54]. 20E induces a rapid increase in the intracellular Ca^2+^ levels [40]; however, the mechanism and consequences were not revealed until recent studies in *H. armigera*. Ca^2+^/calmodulin-dependent protein kinase II (CaMKII) is a serine/threonine-specific protein kinase that is regulated by the Ca^2+^/calmodulin complex [55]. CaMKII expression and phosphorylation increase during metamorphosis in *H. armigera* [56]. 20E regulates phosphorylation of CaMKII at threonine 290, which induces CaMKII translocation into the nucleus. ErGPCR-1 and ErGPCR-2, Gαq, PLC, and Ca^2+^-signaling are involved in 20E-induced CaMKII phosphorylation. RNAi-mediated *CaMKII* knockdown prevents larval-pupal transition and 20E-responsive gene expression. The phosphorylation and nuclear translocation of CaMKII induces the phosphorylation and nuclear export of histone deacetylase 3, thus maintaining USP lysine acetylation at amino acid 303. This modification is necessary for its interaction with EcR to form the transcription complex and for the binding of the EcR-USP complex to EcRE [56]. 20E, through GPCRs, induces intracellular Ca^2+^ release, which causes stromal interaction molecule 1 (STIM1) phosphorylation and aggregation. Aggregated-STIM1 moves toward the plasma membrane to interact with orai1 for Ca^2+^ entry [57]. In turn, orai1 expression is upregulated by 20E [58]. The high levels of 20E switches autophagy to apoptosis in the *H. armigera* midgut by increasing the Ca^2+^ levels in cells [59], thereby inducing apoptosis [60]. Therefore, 20E increases the intracellular Ca^2+^ levels via a store-operated Ca^2+^ entry (SOCE) mechanism.

In addition to triggering rapid increases in intracellular Ca^2+^ levels to activate the PKC pathway, 20E also stimulates a rapid increase in cAMP levels and activates the protein kinase A (PKA) pathway in *H. armigera* [61]. The expression of the catalytic subunit 1 of PKA (PKAC1) increases during metamorphosis, and that *PKAC1* knockdown blocks pupation and represses 20E-responsive gene expression. Through ErGPCR2, 20E regulates PKAC1 phosphorylation and its nuclear translocation. PKAC1 induces the phosphorylation of cAMP response element-binding protein (CREB) at serine 143, which allows it to bind to the cAMP response element (CRE) to enhance 20E-responsive gene transcription. Through ErGPCR2, 20E increases cellular cAMP levels, which induces PKA-mediated CREB -phosphorylation and, in turn, promotes 20E-responsive gene expression. Thus, the 20E-induced PKA/CREB pathway enhances the 20E-induced PKC pathway for gene transcription [61].

## Termination of 20E signaling

The mechanism by which the 20E signal is desensitized remains unclear. A study in *H. armigera* showed that β-arrestin-1 expression levels are markedly increased in tissues during *H. armigera* metamorphosis [62]. Further study showed that in contrast to the 20E-promoted pupation, interference with *Arrb1* (encoding β-arrestin-1) by dsRNA injection into larvae causes advanced pupation and a chimeric larva-pupa phenotype. β-arrestin-1 depletion increases the mRNA levels of 20E-responsive genes, while their levels are decreased by *Arrb1* mRNA overexpression. Following 20E induction, β-arrestin-1 migrates to the cytoplasmic membrane from the cytoplasm to interact with ErGPCR-1. Via ErGPCR1, 20E regulates β-arrestin-1 phosphorylation at serines 170 and 234, and mutation of these residues inhibits 20E-induced β-arrestin-1 migration to the cell membrane. Therefore, via negative feedback mechanism, 20E induces β-arrestin-1 phosphorylation and cell membrane migration, which blocks 20E signaling by the interaction between β-arrestin-1 and ErGPCR-1 [62].

GPCR kinase (GRK)-induced desensitization of 20E-mediated GPCR signaling in the cell membrane was first revealed in *H. armigera* [63]. GRK2 protein levels increase during the metamorphic stage under 20E regulation. *GRK2* knockdown in larvae causes accelerated pupation, an increase in 20E-responsive gene expression, and advanced apoptosis and metamorphosis. 20E induces GRK2 translocation from the cytosol to the cell membrane via 20E-responsive ErGPCR-2. GRK2 is phosphorylated at serine 680 by PKC after induction by 20E, which leads to the translocation of GRK2 to the cell membrane. GRK2 then interacts with ErGPCR-2 and phosphorylates ErGPCR-2 to induce its internalization. Therefore, GRK2 terminates the ErGPCR-2 function in 20E signaling at the cell membrane via a negative feedback mechanism [63].

## The relationship between genomic actions and the nongenomic actions of steroid hormone

The genomic actions of a steroid hormone include that the hormone freely diffuses into cells, binds to its nuclear receptor to form transcription complex, and binds to promoter in DNA to initiate gene transcription. This gene transcription-related pathway is named genomic pathway. The genomic action/pathway occurs in the nuclei after the steroid hormone binding to the nuclear receptor; therefore, this pathway is also known as a nuclear receptor pathway. The genomic actions are relatively slow because the gene transcription and protein translation take time. Whereas, the nongenomic actions of a steroid hormone include the rapid cellular responses, such as calcium influx in seconds, variation of protein phosphorylation, subcellular localization and protein interaction. This rapid cellular response-related pathway is named nongenomic pathway. The nongenomic action/pathway occurs in the cytosol after the steroid hormone binding to the cell membrane receptor; therefore, this pathway is also known as a cell membrane receptor pathway. An intriguing question is the relationship between the genomic action/pathway and the nongenomic action/pathway. From the studies in *H. armigera*, the genomic action/pathway of 20E is regulated by the nongenomic action/pathway, because the 20E-induced rapid calcium increase in cells activates protein kinases [52, 56], therefore promotes EcR-USP transcription complex formation, which initiates gene transcription in 20E pathway finally [64]. Therefore, the 20E signaling pathway is a GPCR-mediated signaling pathway (20E-GPCR pathway).

Another interesting question is whether steroid hormones passively enter cells. An ATP-binding cassette (ABC) protein in the plasma membrane that exports steroids in yeast suggests that similar membrane sorting systems in mammalian cells [65]. In *Drosophila*, ecdysone is released out of cells via ABC protein that functions as an ecdysone transporter [66, 67]. ErGPCR-2 in the lepidopteran *H. armigera* increases 20E entering cells [48]. Recent work suggests that 20E entry into cells is controlled by a 12 transmembrane protein, Ecdysone Importer (EcI), in *Drosophila* [67]. These data suggest that 20E is not passively enter the cell membrane [68]. The recent work in *H. armigera* shows the 20E concentrations are different in various tissues when they are soaked in same hemolymphic 20E concentration [69], suggesting that the different concentrations of 20E in cells induce different gene transcription via EcR, and 20E entry into cells is controlled by some unknown mechanism, which needs further study.

## Function of ecdysone

Ecdysone (E) is the precursor of 20E [70]. Insects do not synthesize steroid precursors, but ingest animal cholesterol or plant phytosterols in the form of food, which is then processed to generate ecdysone. 20E is an ecdysteroid hydroxylated at the 20th carbon in addition to other carbons [71]. Although 20E is the active molting hormone and ecdysone is relatively inactive, E accelerates the metamorphic timing and results in elevation of lethality during metamorphosis in *D. melanogaster*, suggesting both 20E and E are essential for the regulation of metamorphic timing in *D. melanogaster* [72]. Both E and 20E exist in plants to play roles in the plants or participate in the defense of plants against insects [73]. Therefore, the insecticidal activity of the 20E can be developed to control insect pests [74].

## Discussion

GPR30 is suggested as the cell membrane receptor for the steroid hormone estrogen by detection of the binding of fluorescence-labeled estrogen in Cos7 cells [25]. Subtypes of estrogen receptors of GPER are found in human cerebral vascular endothelial cells [75]. DmDopEcR in *Drosophila* is reported to bind the 20E analog [^3^H]-labeled Pon A [30]. DopEcR in *H. armigera* can bind 20E in the cell membrane or as an isolated protein [49]. To prove the GPCRs binding steroid hormone is very difficult. One reason is the difficulty of overexpressing and purifying GPCRs. Another reason is the difficulty of labeling the ligands and detecting ligand binding to the dynamic GPCR proteins [76]. GPCRs undergo highly dynamic structural changes during signal transduction upon the binding of their ligands and interactions with intracellular effectors [77]. For example, ligand binding to beta-adrenergic receptors (ARs) results in a conformational change that activates Gs protein complexes [78]. The cholesterol binding site on the lipid-transmembrane interface of 2-AR has been reproduced in different crystal forms [79]. The cholesterol hot-spots of GPCRs have a microsecond time scale of exchange upon lipid binding; therefore, they might be termed “high occupancy sites” rather than “binding sites” [80]. Therefore, investigation of individual GPCRs using conventional experimental techniques is extremely challenging [81]. A suitable method to detect the dynamic binding of small lipid ligands to GPCRs needs to be established. 20E-EIA detects the binding of GPCRs to 20E directly, presenting an alternative method to that using the isotope-labeled analog Pon A [49].

GPCRs are considered to function not as a single molecule, but in the form of homo- or hetero-oligomers [82]. Some GPCRs can form receptor mosaics via the further assembly of three or more protomers [83]. Oligomerization of GPCRs results in the diversification of receptor signaling [84]. GPCRs exist as an ensemble of temporally interchanging conformations [85]. Although the significance of homo- or hetero-oligomers of GPCRs is unclear, the study of GPCR oligomers might reveal evidence to explain the reported involvement of various GPCRs in the same signal pathways.

20E regulates gene transcription via its nuclear receptor EcR in *H. armigera* [64]. Via the GPCR-mediated nongenomic pathway, 20E regulates the phosphorylation and lysine acetylation of nuclear transcription factors to form the transcription complex, EcR/USP, and the binding to EcRE for further gene transcription; therefore, via the nongenomic pathway, 20E regulates the genomic pathway. 20E signaling might involve several GPCRs, probably depending on GPCR homo- or hetero- oligomerization, their tissue specific distribution, time-related expression patterns, and downstream factors, all of which require further investigation.

20E, which is produced in both insects and plants, such as *Cyanotis vaga*, can be used to disrupt the development of insect pests [86]. Insect ecdysone is converted from dietary cholesterol in the insect prothoracic gland and then processed into 20E in the hemolymph or tissues [87]. 20E can be used as a food additive to help maintain the glucose-lipid balance in humans, without the side effects associated with the use of mammalian hormones [88]. 20E changes energy metabolism to inhibit stem cell proliferation in *Drosophila* [89]. In *H. armigera*, high concentrations of 20E induce Ca^2+^ influx to switch autophagy to apoptosis in the midgut [59, 60]. The identification of GPCRs as 20E cell membrane receptors will improve our understanding of the roles of 20E and its mechanism of action in mammals and in insects.

Approximately 30–35% of marketed drug targets are GPCRs [90–92]. The challenge to achieving full elucidation of the steroid hormone-GPCR pathway is critically dependent on establishing a method to detect the highly dynamic conformations of GPCRs and their interaction with steroids. Recent researches in insects have revealed the axis of the 20E signaling pathway via GPCRs, which will encourage the study of steroid hormone signaling pathways that act via GPCRs. In addition, GPCRs represent targets to develop new types of insecticides.

## Conclusions

**Fig. 1:**
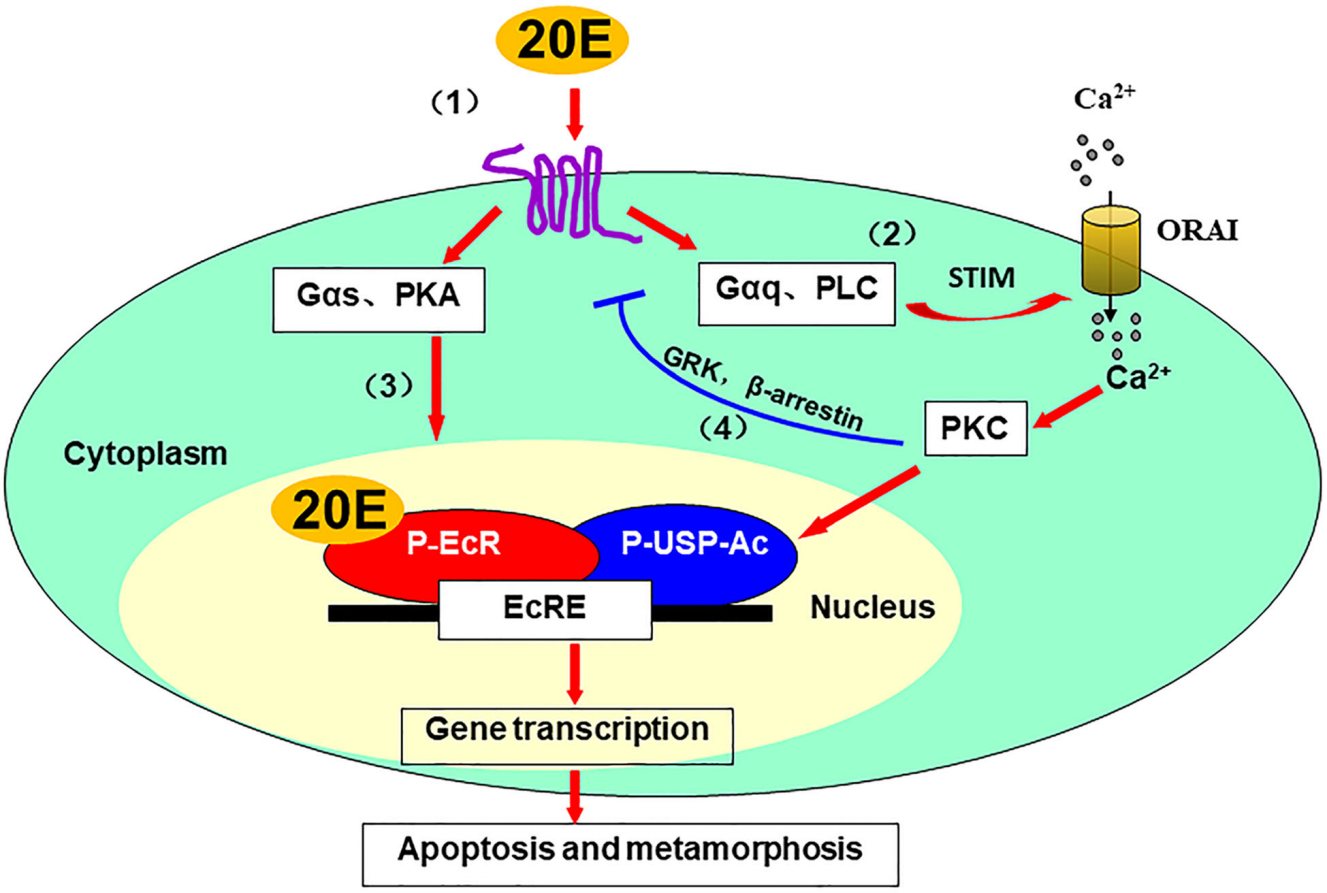
Schematic interpretation of the GPCR-mediated signaling pathway of 20E (20E-GPCR pathway). 20E binds to GPCRs to activate Gαs and Gαq, respectively, to trigger a further signal cascade (1). 20E via Gαq and PLC activates STIM to induce SOCE to activate PKC, which induces USP phosphorylation and acetylation to form the P-EcR/P-USP-Ac transcription complex that promotes 20E pathway gene expression (2). 20E via Gαs and PKA enhances gene transcription (3). 20E regulates β-arrestin-1 and GRK2 phosphorylation and cell membrane migration to induce GPCR internalization or desensitization to terminate 20E signaling via a negative feedback mechanism (4)

Studies of the insect steroid hormone 20E show that GPCRs function as cell membrane receptors of steroid hormones. 20E binds to GPCRs to trigger the PKA and PKC pathways via Gαs and Gαq, respectively. 20E induces calcium influx by SOCE via Gαq, PLC, STIM, and Orai-signaling. The increased level of calcium acts as a secondary messenger to induce protein phosphorylation and acetylation to regulate the nuclear receptor transcriptional complex formation for gene transcription and apoptosis. 20E, via the PKA pathway, enhances the PKC pathway. 20E signaling is terminated by desensitization of GPCRs via a negative feedback mechanism. A GPCR-mediated signaling pathway of 20E (20E-GPCR pathway) has been elucidated, which opens a door to further study of steroid hormone signaling via GPCRs (Fig. 1). There are still many questions need to be answered, such as the facilitated- or passive-entry of steroid hormones into cells, the homo- or hetero- oligomerization of GPCRs, and several GPCRs function in a same pathway.

## Data Availability

Not applicable.

## Abbreviations

GPCR: G protein-coupled receptors
GPER1: G protein-coupled estrogen receptor 1
DopEcR: Dopamine/ecdysteroid receptor
20E: 20-hydroxyecdysone
EcR: Ecdysone receptor
USP/RXR: Ultraspiracle protein/retinoid X receptor
ErGPCR: 20E-responsive GPCR
ER: Estrogen receptors
AR: Androgen receptors
PLC: Phospholipase C
STIM1: Stromal interaction molecule 1
SOCE: Store-operated Ca^2+^ entry
PKA: Protein kinase A
PKC: Protein kinase C
Gαq: G protein alpha q subunit
cAMP: Cyclic adenosine monophosphate
[^3^H] Pon A: [^3^H] ponasterone A
GRK: GPCR kinase
CREB: camp response element-binding protein
HaEpi cells: *H. armigera* epidermal cell line
